# Investigation of the mechanical behavior of rock-like material with two flaws subjected to biaxial compression

**DOI:** 10.1038/s41598-024-64709-x

**Published:** 2024-06-19

**Authors:** Shuailong Lian, Wen Wan, Yanlin Zhao, Qiuhong Wu, Can Du

**Affiliations:** 1 School of Resources Environment and Safety Engineering, Hunan University of Science and Technology, Xiangtan, 411201 Hunan China; 2 College of Civil Engineering, Guizhou University, Guiyang, 550025 Guizhou China

**Keywords:** Rock-like material, Biaxial compression, Propagation and penetration, Peak strength, Environmental sciences, Engineering

## Abstract

The biaxial compression experiments of rock-like materials with two flaws are carried out under different flaw inclination angle, rock bridge ligament angle, lateral stress. The experimental studies show that crack propagation modes of rock-like material are as follows: wing crack through mode (Y mode), shear crack through mode (J mode), mixed crack through mode (wing shear JY mode), longitudinal extension of crack and transverse shear splitting. prefabricated fractured rock specimens have experienced the closing stage of prefabricated fractures, the elastic deformation stage, the generation and expansion of cracks (or plastic strengthening), and the residual loading stage. The peak strength of the specimen is increases with the increase of flaw inclination angle and lateral stress. With the increase of the rock bridge ligament angle, the failure of the rock bridge region changes from the shear crack failure to composite failure of shear crack and the wing type tensile crack failure, and then to the wing crack failure. With the increase of the lateral pressure, the failure of the specimen changes from the wing type tensile crack failure to the wing type and shear crack failure, and then to shear crack failure. The flaw inclination angle mainly changes the form of crack growth but does not effect on the failure modes. The counting number of acoustic emission events at the center of the sample is relative large, indicating that the cacks appear in the part of the rock bridge firstly. With the increasing of loads, the cracks of the rock bridge expanding constantly and connecting finally. The changes of acoustic emission event counts is consistent with the macroscopic damage form obtained from the experiments.

## Introduction

Under the influence of long-term strata tectonic movement, micro-cracks and mesoscopic internal defects are bound to be bred in rock mass, which makes them develop into macroscopic joint fractures, faults and other different scales and different types of defects in geological environment. The extension of adjacent joints and fissures in engineering rock mass results in instability and failure of rock mass, which leads to geological disasters and engineering problems such as rock collapse, land slide and water inrush in tunnels, and causes heavy economic losses and casualties. Therefore, it is of great significance and further-reaching the influence to study the mode of joint expansion and connection in the fractured rock mass.

To study the mechanical characteristics of rocks with discontinuous joints, many theoretical and experimental studies were conducted on defective rock materials under uniaxial compression, these studies provide a comprehensive understanding of the strength, deformation, and fracture breakdown patterns of defective rock materials^1–10^. At present, the research of prefabricated flaws rock specimens is mainly focused on several key influencing factors under biaxial compression such as prefabricated fracture arrangement density, flaw inclination angle, rock bridge ligament angle and lateral stress. However, most of the studies focus on the influence of different factors on the axial peak stress according to many existing research results^6–19^. For example, the axial peak strength increased first and then decreased with the increase of lateral stress^20–26^. However, the studies on crack propagation of rock specimens are still rare under lateral stress, and the explain about this phenomenon are rare from the perspective of failure mechanism^27–35^. As far as the research method of crack propagation failure test is concerned, with the development of software technology, many scholars choose image analysis software combined with digital image correlation technology and acoustic emission localization technology at home and abroad^36–40^. It is observed in the experiment that the crack is mainly initiated by the crack near the tip of the prefabricated flaw, and the crack is divided into tension-wing cracks. There are many forms of compression-shear secondary crack and wing crack reverse crack, but scholars are pay more attention to the tensile failure of wing crack, there are few researches on shear crack and its failure mechanism^41–53^. The above research is of some significance to the crack expansion and breakdown of brittle rock materials, but it is rare investigation on the crack expansion of rock-like specimens subject to the biaxial compression conditions.

Based on the above studies, scholars have conducted a lot of studies on the propagation of prefabricated cracks in brittle rock materials under uniaxial and biaxial compression^54–58^, but there are few studies on the influence of lateral stress on the crack propagation process of rock specimens, and most of studies mainly focus on the fracture during the test of fractured rock materials. Moreover, there is little research on shear crack and its damage mechanism. Therefore, biaxial compression tests were carried out on rock-like materials containing two prefabricated flaws, and the influences of prefabricated flaw inclination angle, rock bridge ligament angle and lateral stress on the strength and deformation characteristics of test samples were studied. The influences of prefabricated flaw inclination angle, rock bridge ligament angle and lateral stress on crack propagation were analyzed, and the detailed processes of initiation, propagation and merger of new cracks and existing defects were revealed. Furthermore, this study also reveal the relationship between crack propagation and stress characteristics by combined with acoustic emission monitoring system.

## Study on fracture mechanical properties of fissure rock mass

### Analysis of the rock fissure fracture strength

**Figure 1:**
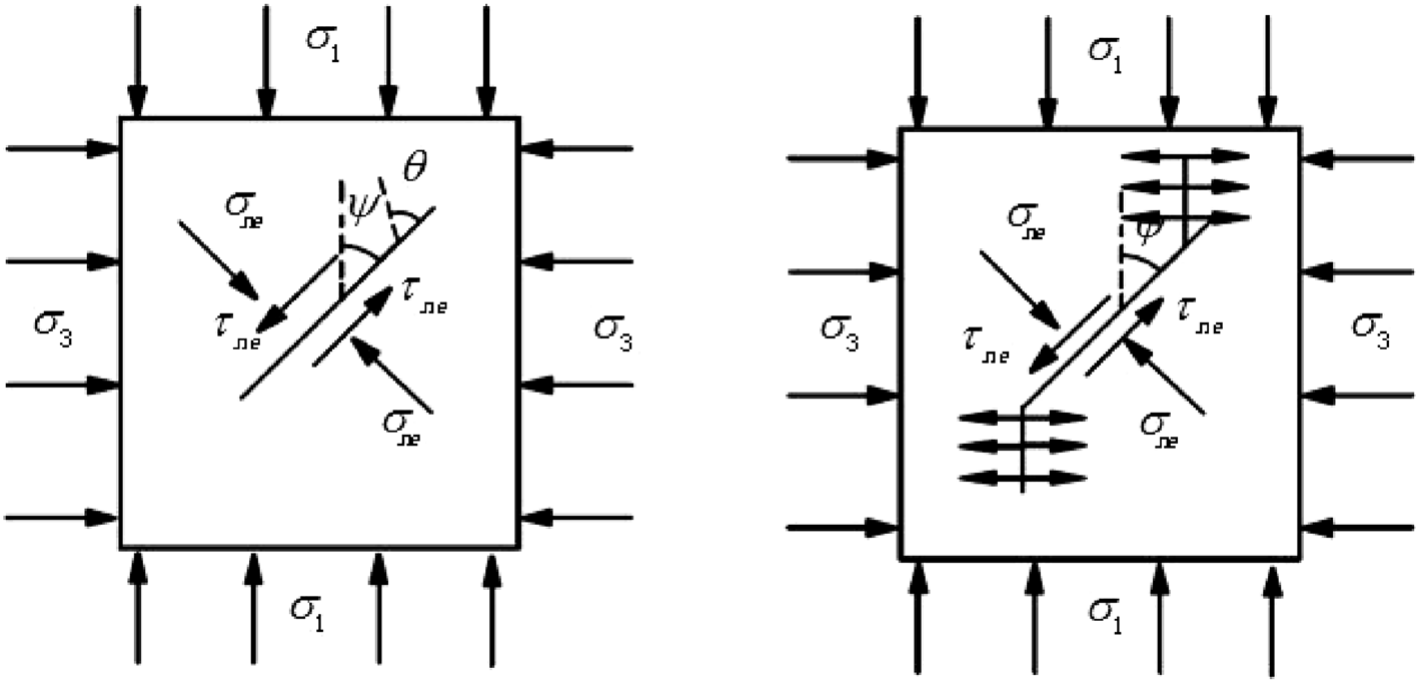
Initiation and expand of cracks under the pressure of the axial and shear stress.

Most of the rock bodies are assigned contain internal defects such as macro or onlookers cracks in the nature. The process of rock damage is essentially to study the process of cracking, expansion and penetration of the internal cracks under the action of external force. In the state of surrounding multiple force, the crack starts to the maximum tensile stress from the direction approximately perpendicular, expanding and broken with the wing crack at the form of tension, as shown in the Fig. 1.

The upper tangential and normal stress of the fracture surface are respectively:

1 $$\tau_{ne} = \frac{{\sigma_{1} - \sigma_{3} }}{2}\sin 2\psi$$

2 $$\sigma_{ne} = \sigma_{1} \sin^{2} \psi + \sigma_{3} \cos^{2} \psi$$

The shear stress $\tau_{ne}$ will cause mutual sliding between the fracture surfaces, and the crack is compacted as the axial stress increasing, the friction coefficient is $f$, the friction resistance is $f\sigma_{ne}$ between the friction surfaces, the shear driving force of the fracture slip is $T_{e}$.

3 $$T_{ne} = \tau_{ne} - f\sigma_{ne} = \frac{{\sigma_{1} - \sigma_{3} }}{2}\sin 2\psi - f\sigma_{ne}$$

According to the fracture mechanics theory, the stress strength factor of the crack tip is:

4 $$K_{{\text{I}}} = - \sigma_{{{\text{ne}}}} \sqrt {\pi a}$$

5 $$K_{{{\text{II}}}} = T_{{\text{e}}} \sqrt {\pi a}$$

6 $$K_{{\text{I}}} = - (\sigma_{1} {\text{sin}}^{2} \alpha + \sigma_{3} \cos^{2} \beta )\sqrt {\pi a}$$

7 $$K_{{\text{I}}} = \left( { - \frac{{\sigma_{1} + \sigma_{3} }}{{2}} + \frac{{\sigma_{1} - \sigma_{3} }}{{2}}{\text{cos2}}\psi } \right)\sqrt {\pi a}$$

8 $$\begin{aligned} K_{{{\text{II}}}} & = \frac{{\sigma_{1} - \sigma_{3} }}{2}{\text{sin}}2\psi - f\sigma_{ne} \\ & = \left( {\frac{{\sigma_{1} - \sigma_{3} }}{2}\sin 2\psi + f\frac{{\sigma_{1} + \sigma_{3} }}{2} - f\frac{{\sigma_{1} - \sigma_{3} }}{2}\cos 2\psi } \right)\sqrt {\pi a} \\ \end{aligned}$$

where, $\sigma_{1}$ and $\sigma_{2}$ are maximum and minimum main stress, $\psi$ is angle between maximum main stress and crack surface; a is the half crack long.

### Cracking analysis of the shear crack under the maximum circumferential stress criterion

According to the study of rock fracture mechanics, the maximum peripheral stress criterion is consistent to the test conclusion.

Establishing polar coordinates $(r,\theta )$ with the crack tip as the origin, the stress $\sigma_{\theta }$ can be expressed as:

9 $$\sigma_{\theta } = \frac{3}{2}\frac{{\tau_{e} \sqrt {\pi a} }}{{\sqrt {2\pi r} }}\sin \theta \cos \frac{\theta }{2}$$

As shown in the Fig. 1, the crack tip stress strength factor can be expressed as:

10 $$K_{{\text{I}}} = \frac{3}{2}T_{e} \sqrt {\pi a} \sin \theta \cos \frac{\theta }{2}$$

Under the maximum circumferential stress criterion, the initial crack of the rock extends in the direction of the maximum circumferential stress, and it is satisfied:

11 $$\frac{{\partial \sigma_{\theta } }}{\partial \theta } = 0$$

From Eqs. (9) and (10), the crack opening angle is $\theta$ = 70.5°, and the replacement Eq. (10) can obtain the initial crack starting factor as

12 $$K_{{\text{I}}} = \frac{2\sqrt 3 }{3}T_{e} \sqrt {\pi a}$$

### Expansion analysis of crack under stress

The Griffith energy criterion has always been an important theoretical basis for the field of fracture mechanics, by analyzing the crack extension from the perspective of the Griffith energy criterion. The criterion introduces the inevitable energy consumption of crack expansion, and the crack surface area is bound to increase in the process of crack expansion. If the crack indicates that the energy is $\Gamma$, When the crack expansion increases the area is $\Delta S$, the energy consumption required is $\Delta \Gamma$. It is available:

13 $$\Delta \Gamma = R \cdot \Delta S\;{\text{or}}\;R = \frac{\Delta \Gamma }{{\Delta S}}$$

Take the limit of $\Delta S$ is variable to:

14 $$R = \mathop {\lim }\limits_{\Delta S \to \infty } \frac{\Delta \Gamma }{{\Delta S}}$$

R is the energy needed for crack expansion unit area, is called the extension resistance of crack.

Therefore, it must be provide certain power to make crack expansion, and the power provided by crack unit area system is G, to meet the conditions of crack expansion:

15 $$G \ge R$$

Equation (15) is the Griffith energy criterion: per crack expansion unit area, the power G provided by the system must be greater than or equal to the crack expansion resistance. Where, $G > R$ the crack will be in the state of accelerated expansion. Then, $G < R$, the crack will not occur expansion, $G = R$, the crack can be called a sub-critical extension.

The research of fracture mechanics of rock shows that the rock mass crack in a certain medium of stress environment, the stress strength factor of the crack tip $K_{{\text{I}}}$ does not reach its fracture toughness $K_{JC}$, that is, the rock crack will be in a stable and quasi-static expansion state, that is, the subcritical expansion state described above. $K_{{\text{I}}}$ can be used as an important parameter for the analysis of crack expansion during the stress corrosion of crack rock. The corresponding lower limit of stress corrosion is recorded $K_{0}$, when $K_{{\text{I}}}$ < $K_{0}$, the crack will not expand, and $K_{0} < K_{{\text{I}}} < K_{JC}$, the crack will expand, namely the rock rheological disturbance effect. The rock mass engineering leading to instability damage as the rock mass crack gradually expanding. Therefore, the study of crack expansion mechanism is the fracture mechanism of rock engineering.

## Specimen preparation and testing

### Specimen preparation

**Figure 2:**
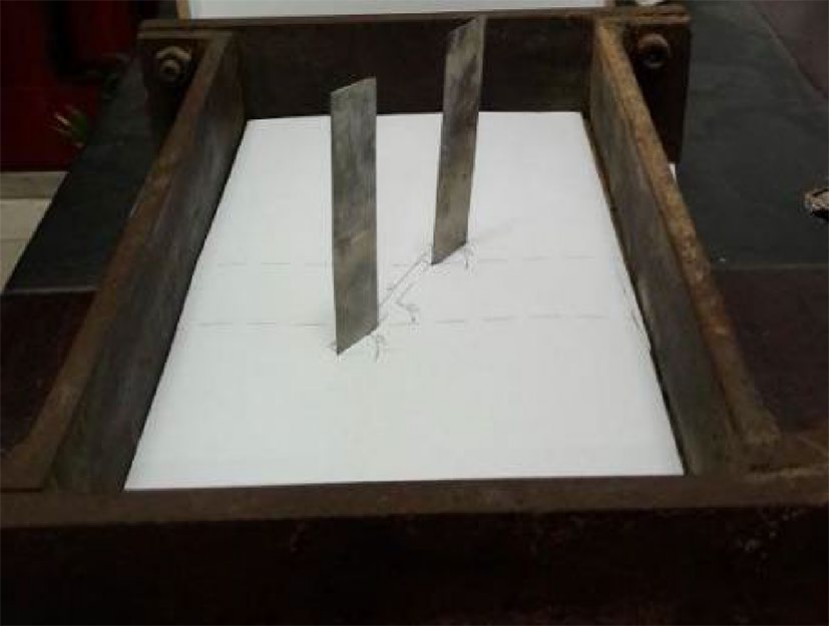
Mold used to produce of specimens.

**Figure 3:**
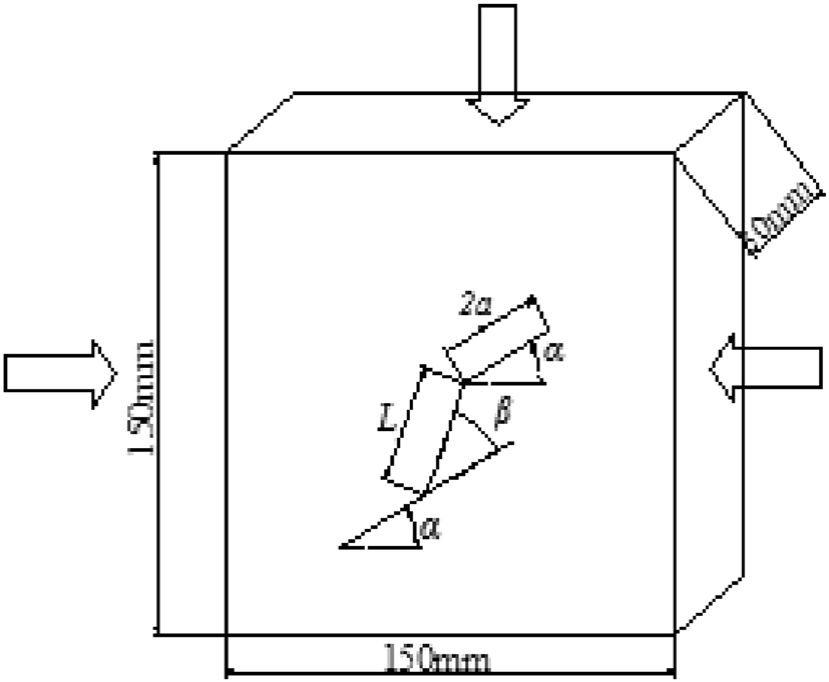
Geometry of specimen containing two flaws.

The rock-like specimens were made of a mixture of ordinary Portland cement, sand and water. The sand was a mixture of 60% fine (0.1–0.25 mm) and 40% medium (0.25–0.40 mm) sands. First, the cement, sand and water were mixed in a blender at a ratio of 2:1:0.4 by weight to produce the cement mortar^59^. Then, the mortar was poured into a mold with internal dimensions 150 × 150 × 30 mm^60^. The mold consisted of four steel plates on the sides and two plastic plates on the top and bottom (Fig. 2). The two plastic plates were identical with two slots. The pre-exist flaws in the specimens were created by inserting two thin metallic sheets (20 mm wide and 0.3 mm thick) into the slots and through the mortar, resulting in a flaw length (2*a*) of 20 mm and an aperture (*b*) of 0.3 mm. The position and orientation of the slots were varied to give different combinations of flaw inclination angle (α), rock bridge ligament angle (β), and bridge ligament length (*L*) as shown in Fig. 3. In this paper, the term ‘‘flaw’’ is used to describe the pre-exist fissure or crack and the term ‘‘crack’’ is adopted to describe the new fracture or failure during loading. In this study, α was set to 30°, 45° and 60°, respectively, β varied from 0° to 90° and *L* was set to 2*a* (20 mm). The mold with the fresh cement mortar was vibrated for 10 min using a vibrating table and then stored in a room at a temperature of 20 °C for 24 h. Then, the specimens were molded and put in water for curing at a temperature of 20 °C for 28 days. After the curing, the surfaces of the specimens were poished with coarse (grit #60) and then fine (grit #200) sandpapers before tested. Considering the variation related to specimen fabrication, there are four specimens were prepared for each condition.

### Testing

**Figure 4:**
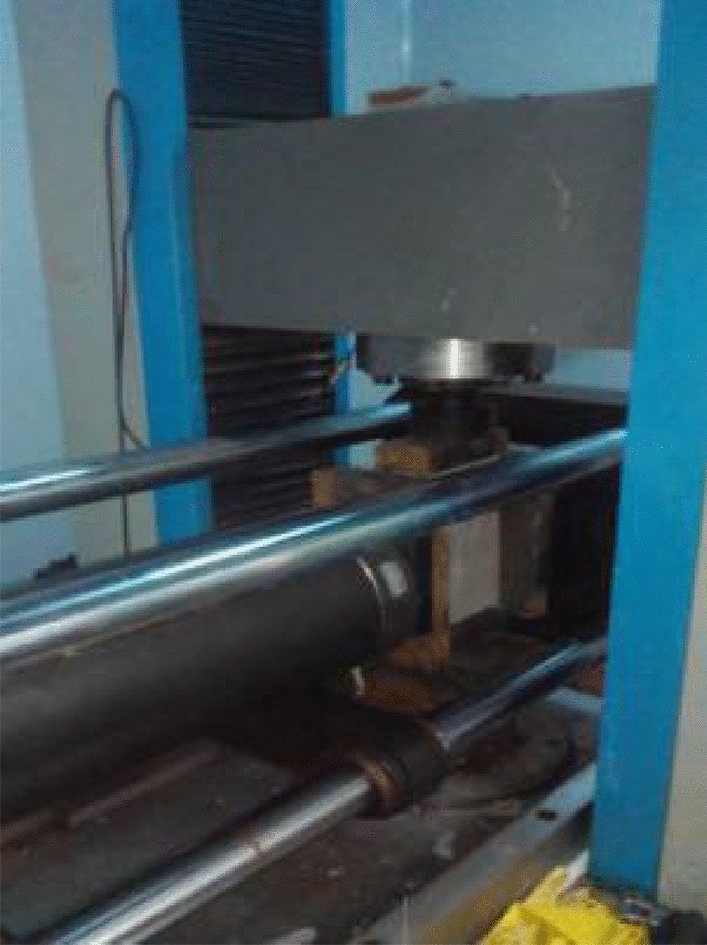
Biaxial loading tests.

**Figure 5:**
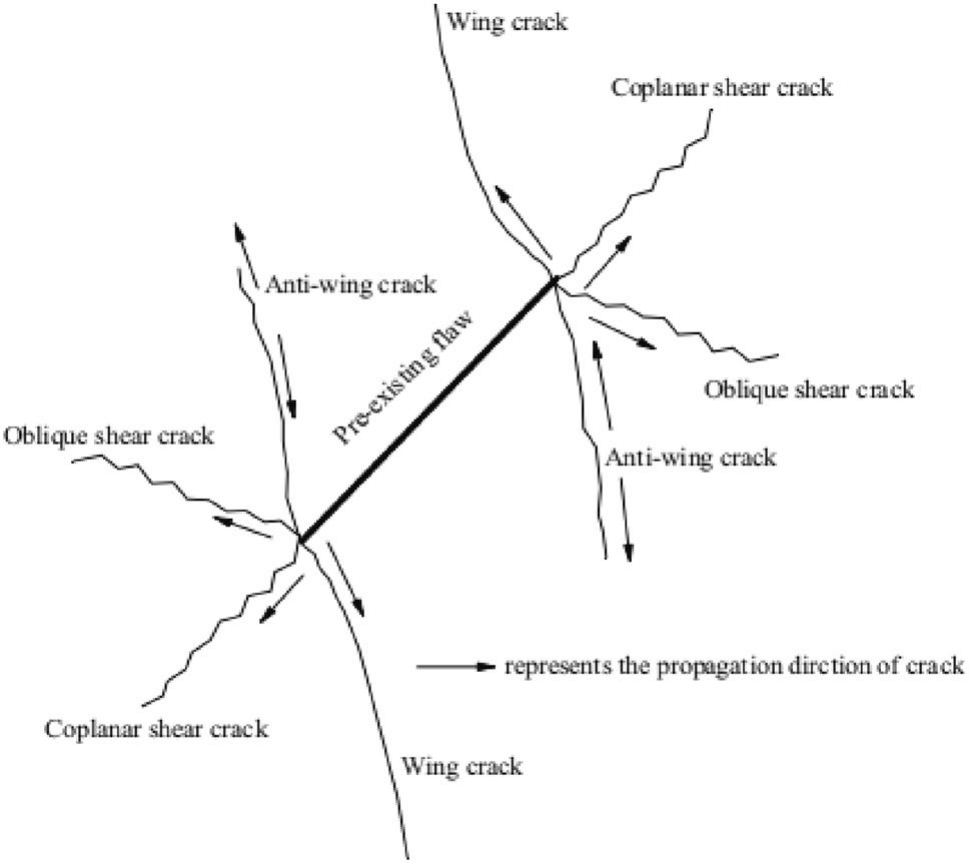
Cracks types observed in the flawed specimens.

Biaxial compression testing was performed on the RYL600 to the defective specimen and the intact specimen. In order to reduce the end effect, two polythene sheets coated with butter were placed between the end of the specimen and the loading platen. The upper platen was placed on a low friction linear bearing so that it can move freely in the horizontal direction. The test setup and the details of the specimens under loading were shown in Fig. 4. Loading was applied by displacement control at a low rate of 0.002 mm/s. Firstly, it was loaded at 100 N/S loading rate to a predetermined lateral pressure (0 MPa, 0.5 MPa, 1 MPa, 2 Mpa) and then applied the vertical loading rate pressure of 150 N/s until the specimens was destroyed. It aims to improve the observation effect of crack formation, development and coalescence. During the progress of test, the vertical load and displacement were measured simultaneously using the data acquisition system of the RYL600. The AE probe of the acoustic emission device places the AE probe device according to the predetermined position (30,30), (30,120) (120,30), (120,120) in the test scheme, places the rock specimen of the audio emission device on the RYL-600 shear rheometer for loading, and starts the collection of the acoustic emission events. A digital camcorder and a high-speed camera were also used to monitor the crack initiation, propagation and coalescence. The high-speed digital camera was used to capture the instants of crack initiation and coalescence with a sampling rate of 10,000 fps (frames per second), while the digital camcorder recorded the whole process. The specimen was loaded until it failed or cracks had coalesced. During the testing, the vertical load and displacement were measured every 0.5 s. The test specimens and its name number were shown in Table 1 (Fig. 5).

**Table 1:** Details of all tested rock-like specimens.

α (°)	β (°)	Lateral stress 0 MPa	Lateral stress 0.5 MPa	Lateral stress 1.0 MPa	Lateral stress 2.0 MPa
30°	0°	M01	M02	M03	M04
	30°	M05	M06	M07	M08
	60°	M09	M010	M011	M12
	90°	M13	M14	M15	M16
45°	0°	M17	M18	M19	M20
	30°	M21	M22	M23	M24
	60°	M25	M26	M27	M28
	90°	M29	M30	M31	M32
60°	0°	M33	M34	M35	M36
	30°	M37	M38	M39	M40
	60°	M41	M42	M43	M44
	90°	M45	M46	M47	M48

## Result and discussion

### Stress–strain curve for the tested rock specimens under biaxial compression

**Figure 6:**
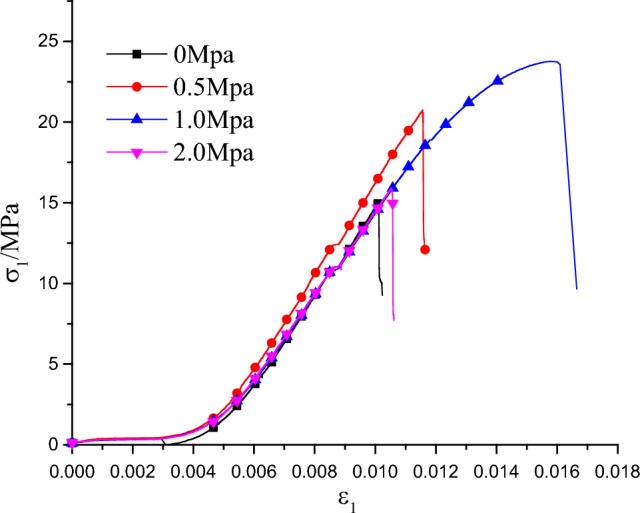
Stress–strain curve with 30° flaw inclination angle and 60° rock bridge ligament angle under different lateral pressures.

Figure 6 shows the axial stress–strain curve with a prefabricated flaw inclination angle of 30° and a rock bridge ligament angle of 60° (i.e. M09, M10, M11, M12 specimens were shown in Table 1) at different lateral stress.

**Figure 7:**
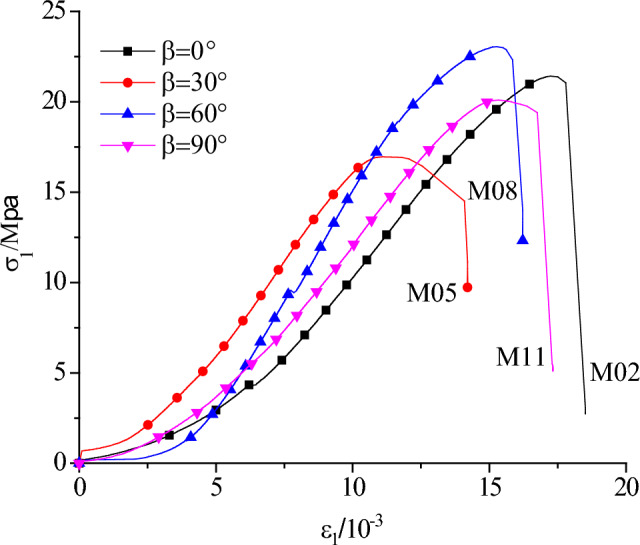
Axial stress strain curve of 30° flaw inclination angle and 0.5 MPa lateral pressures.

Figure 7 shows the lateral stress is 0.5 Mpa, prefabricated flaw inclination angle is 30° (i.e. M02, M06, M10, M14 specimens were shown in Table 1), including the axial stress–strain curve of different rock bridge ligament angle specimens.

The overall curve is roughly S type, the different rock bridge ligament angle has not obvious effect on the axial stress of the specimen under the same lateral stress and flaw inclination angle, while the different lateral stress has a significant impact on the axial stress of the specimens. Since the axial stress of the precast crack test specimens is mainly concentrated between the rock bridge of the crack during the loading. As the axial stress increasing, the crack expands outward until the tested specimens is through, we can see obvious stress drop of the tested specimens after reaching the peak stress. Although the internal structure is damaged to a certain extent after the crack penetration, the friction bond between the crack surfaces has a certain residual strength, and the tested specimens can still basically maintain the overall shape and bear a certain stress.

The axial stress–strain curve of the test specimens under the biaxial compression condition is the plastic–elastic–plastic property, and the curve shows S-shape roughly. According to the Griffith energy criterion, it is inevitably consumes energy every stress drop during the biaxial compression, the test specimens would create new cracks and expand from the macroscopic aspects.

**Figure 8:**
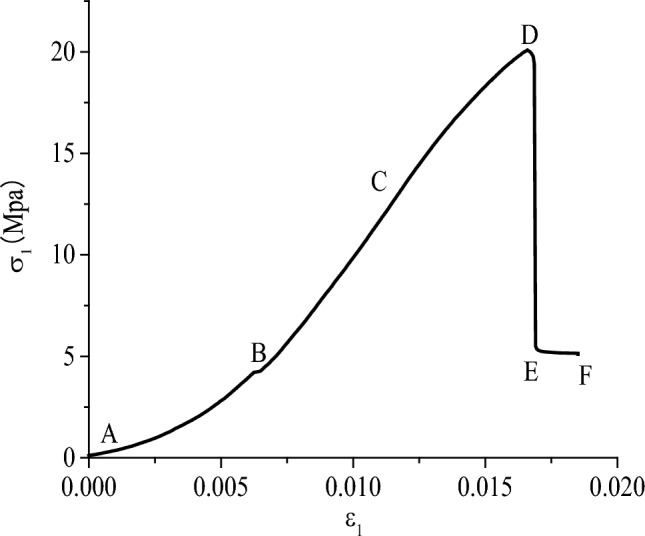
Axial stress Strain curve of 30° × 90° specimens and 0.5 MPa lateral stress.

In order to describe the crack expansion and damage of axial compression of prefabricated double flaw rock specimens, the full stress–strain curve of M14 specimens (i.e. 0.5 Mpa lateral stress, 30°flaw inclination angle and 90° rock bridge ligament angle) was analyzed in the Fig. 8. Axial stress–strain curve of the M14 specimens, as shown in the Fig. 8. The stress–strain curve roughly went through the following four stages: the closing phase of the prefabricated flaw and the internal gap of the rock, the elastic deformation stage, the crack generation and expansion (or shaping reinforcement) stage, and the residual bearing stage.

The AB section shows that the stress–strain curve was concave at the stage of fracture closure, which is nonlinear deformation and without macroscopic crack. The prefabricated cracks and the original open structural surface or small cracks of the test specimens were gradually compacted. The test parts were mainly reflected in vertical strong plastic deformation and less lateral expansion. Due to the low strength of the test part, the strain had a obvious changes in this stage, while the strain is rare little for some rock specimens with high hardness.

The BC section is an elastic deformation stage, which the stress–strain curve is linearly deformed and there is no macroscopic cracks. After the axial stress of the specimens continues to increase after the AB stage, the crack is further compacted and further compressed in volume, mainly reflected in elastic deformation and subject to Hooker’s law.

The CD section shows the stage of crack generation and propagation (plastic strengthening), in which the stress–strain curve deviates from the straight line from point C, and the specimens begins to produce cracks and expand gradually, and the volume of the specimen changes from compression to expansion. The axial strain rate began to increase. It finally reaches point D (peak strength) with the increasing of axial stress.

The DF section is the residual bearing stage. The axial stress is large as soon as it reaches the peak strength of the specimens. After falling (DE section), the specimen immediately appears macroscopic cracks, and roughly through the whole specimens. At this time, the specimens did not appear a large area of damage, and still maintain the basic shape of the specimens. This is because although the axial stress has reached the internal structure of the specimens, the friction bonding between the crack surfaces still has some residual strength and be able to bear a certain load.

If the axial compression continues to increase, the stress will follow the strain. With increasing and small nonlinear increasing, the stress drops rapidly, the specimen loses the bearing capacity completely and reaches the complete failure. In view of the expansion law of the crack and the peak stress, in order to better observe and analyze the crack penetration form, the stress is no longer continued to ensure that the specimens does not have large area failure and collapse.

### Crack coalescence patterns for rock-like specimens subject to biaxial compression

In this paper, all the possible cracks are divided into two common basic types to better describe the cracks form during the process of the test: wing crack and shear crack. The shear cracks can be subdivided into coplanar shear cracks, coplanar and oblique shear cracks due to the dislocation of secondary cracks caused by the axial loading, as shown in the Fig. 5.

The crack propagation and crack morphology of this experiment were as follows: with the increasing of axial stress, the prefabricated flaw were closed and compacted rapidly. Firstly, the airfoil cracks begin to appear at the outer tip (or inner tip) of the prefabricated flaw. With the increasing of axial stress, the crack of wing propagates around the specimens at the form of curve. The wing crack was usually similar to tension crack, and the debris around the crack was less, and the shear crack appearing as the stress increasing, there is a slight dislocation between the two flaws. Then the coplanar shear cracks or oblique shear cracks was produced and propagates outward along the irregular shape. The crack was often realized as the mutual dislocation of the crack, and there were some clastic materials such as thin particles around the crack. With the loads increasing to the peak strength, the failure mode of the specimen was usually extended to the instability of the specimen around the specimen by the airfoil tensile crack or shear crack.

Biaxial compression test was carried out for prefabricated double flaw rock specimens. The fissure inclination angle is 30°, 45°, 60°, respectively, and 0°, 30°, 45°, 60°, 90° respectively. Twelve groups of different types of specimens were under preload of four groups of different lateral stress with axial stress loading until the specimens reaches the peak stress. Due to the complex and various forms of test parts failure crack, according to the test results of different macro fracture types, the crack expansion mode was mainly divided into the following types: wing crack through mode (Y type), shear crack through mode (J type), mixed crack through mode (wing + shear type, JY type), and some tested specimens were not connected in the rock bridge between the precast cracks: crack longitudinal extension and transverse shear splitting. The specific basic several crack expansion modes and the corresponding test results were shown in Tables 2 and 3.

**Table 2:**
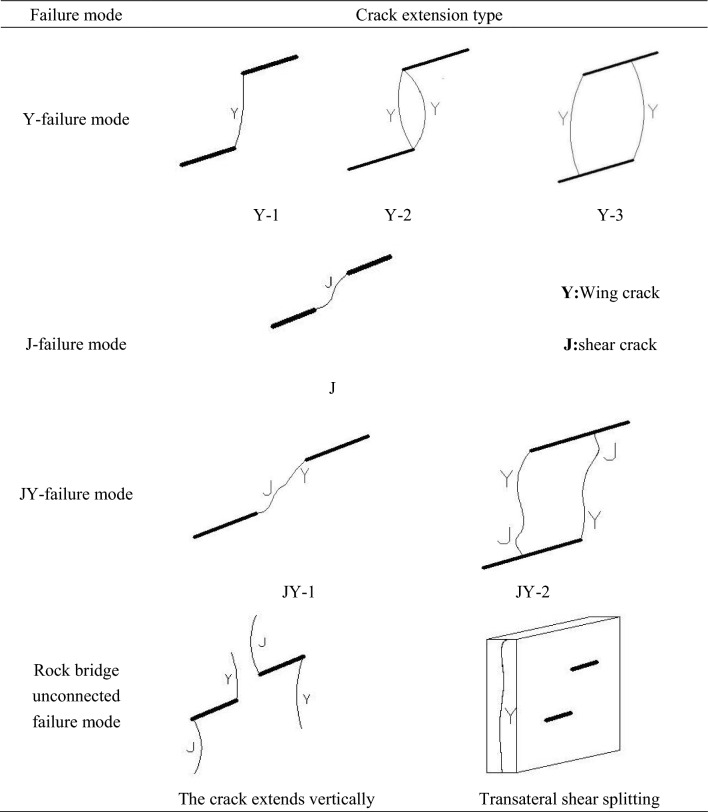
Modes of cracks coalescence.

**Table 3:** The failure mode corresponding to the test result.

Failure mode	Specimens
Y-1	M09, 10, 23, 26, 33, 34, 37, 38, 41
Y-2	M22, 25, 29, 39, 42, 43
Y-3	M13, 14, 16, 31, 45, 46
J	M03, 06, 18, 19
JY-1	M11, 21
JY-2	M15, 28
The crack extends vertically	M01, 02, 05, 07, 17, 30, 35, 47
Transateral shear splitting	M04, 08, 12, 16, 20, 24, 27, 32, 36, 40, 44, 48

Type Y-1 penetration refers to the tensile fracture from the inner tip of one prefabricated flaw. With the continuous increasing of axial stress, the inner tip extends to the inner tip of the other airfoil crack until the rock bridge between the two prefabricated flaw connects with airfoil cracks, sometimes from the center of the bridge to the inner tip of the two prefabricated flaw. The specific expression is that the airfoil crack formed by an approximate smooth curve path runs between the double cracks, connecting the tips of the two prefabricated flaw, respectively (Table 4f,g,i–k).

**Table 4:**
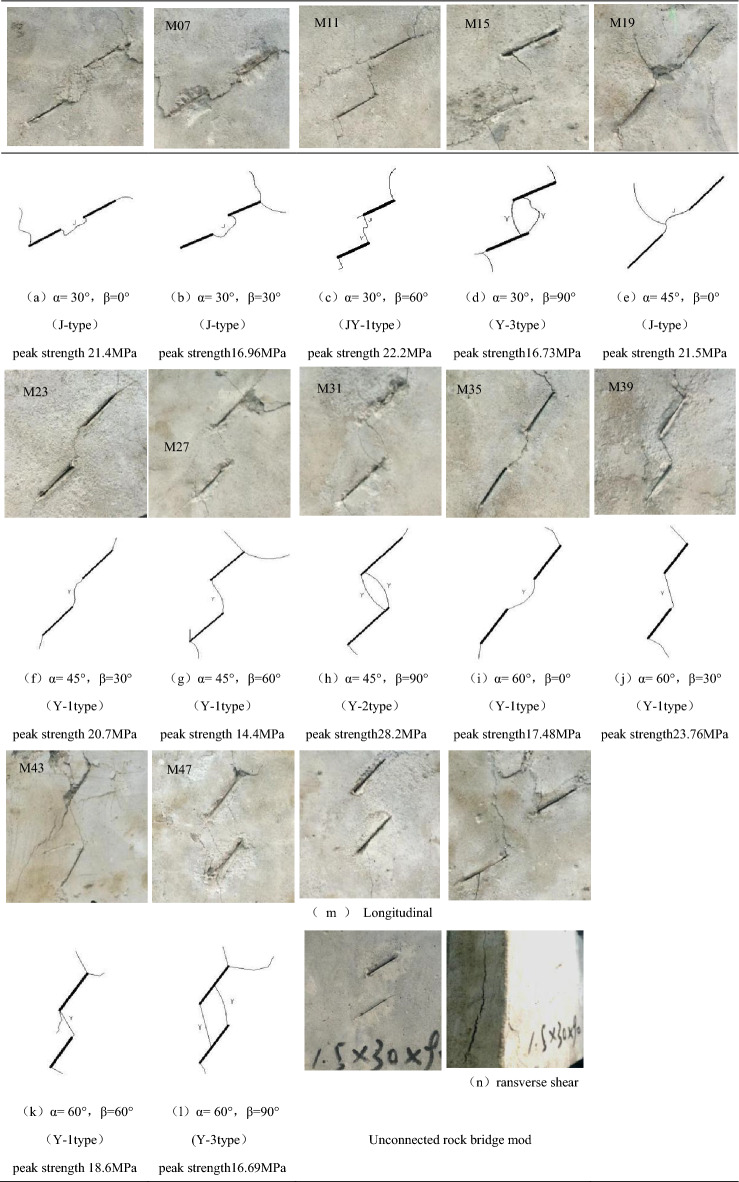
Crack coalescence patterns (Y: Wing crack; J: Shear crack).

Type Y-2 penetration refers to the opening from the inner edge of one prefabricated flaw, extending to the inner tip of the other prefabricated flaw, but unlike the Y-1 penetration, two airfoil cracks form between the two airfoil cracks simultaneously or slightly lag, near the inner tip of the two prefabricated cracks, forming an approximate "fish eye" shape (Table 4h α = 45°, β = 90°).

Type Y-3 penetration is the opening from the inner edge of one prefabricated flaw, extending towards the outer tip of the other, simultaneously or a little, near the edge of the other, until the final two airfoil cracks are closed with two precast cracks, forming a similar "parallelilateral" or "trapezoidal" shape (Table 4d,l).

Shear crack through mode (type J) means that small airfoil cracks occurred initially at the tip of the prefabricated flaw, but then with increasing axial stress, the airfoil cracks were no longer further extended but near them, resulting in shear cracks, either in the inner tip of both prefabricated flaw to some area of the bridge, from the inner tip of the prefabricated flaw to the inner tip of the other. The specific expression is that a shear crack of an irregular curve path connects between the two cracks, connecting the two tips of the two prefabricated flaw. (Table 4a,b,e).

The mixed crack penetration mode (wing + shear type, JY type) can be divided into two different penetration modes.

Type JY-1 penetration means that an airfoil crack cracks and extends from the inner tip of one prefabricated flaw, while or slightly behind the inner tip of the other, and connects in an area of the rock bridge. (Table 4c α = 30°, β = 60°).

Type JY-2 penetration refers to the two airfoil cracks begin to crack and expand at the inner tip of the two prefabricated flaw, to approximate the path of the smooth curve, simultaneously or slightly lag, the outer tip of the two precast cracks began to produce shear cracks and expand, and the two prefabricated flaw were connected in a certain area of the rock bridge. The specific expression form is similar to the crack shape of the Y-3 through type, the difference was that the JY-2 through crack was composed of the wing + shear type cracks.

In this test, there are also some errors of the test part, including the uneven texture and the end effect, causing the cracks during the loading process, the cracks were not successfully connected at the rock bridge between the two precast cracks of the experiment specimens but other forms of crack expansion. It can be roughly summarized into two forms.

The first crack longitudinal extension refers to the beginning to produce airfoil or shear crack at the inner or outer tip of the precast crack, but with the increasing of axial stress, the crack do not expand for the prefabricated flaw, but usually around the longitudinal direction until the test piece is damaged, so the l crack did not completed between the two prefabricated flaw (Table 4m).

The second transverse shear splitting refers to the essential change of the specimen damage form when the loaded lateral pressure reaches a certain degree. As the axial pressure gradually increases, the crack starts from the end position of the experiment specimens edge, and the whole section is then under the shear force until the internal structure of the experiment specimens fails, and the transverse shear splitting occurs in the cross-section direction. Because the whole failure mode has changed, so the experiment specimens failure will no longer be affected by the prefabricated flaw, the connection between the prefabricated flaw cannot be realized (Table 4n).

As shown in Table 4, There are three kinds of crack through modes of rock bridge obliquity and different lateral pressure angles different flaw inclination angles under biaxial compression: wing crack through mode (Y-type), shear crack through mode (J-type) and mixed crack through mode (JY-type). At the same time, a few specimens did not break through in the rock bridge between the flaws. When the lateral pressure increases to a certain extent, the prefabricated flaw would no longer play a decisive role, and the failure form of the specimens would be changed to the lateral shear splitting along the cross section.

### Strength

**Figure 9:**
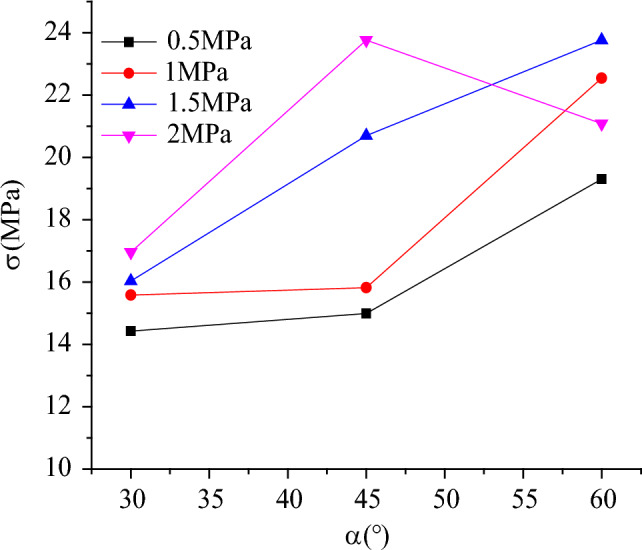
Variation of peak strength with fissure inclination under different lateral stress.

The variation of peak strength with flaw inclination angle under different lateral stress was shown in the Fig. 9. It can be seen from the Fig. 9 that the peak strength increases with the increasing of flaw inclination angle. When the lateral stress is 0 MPa, The flaw inclination angle increased from 30° to 45°, the peak strength increased from 14.43 to 14.99 MPa, and the increase was smaller (increase amplitude was 3.8%). When the flaw inclination angle increased from 45° to 60°, the peak strength increased from 14.99 to 19.3 MPa (32%). In the case of confining pressure, the peak strength increases by 38.8%. When the confining pressure is 0.5 MPa, the flaw inclination angle increases from 30° to 45°, and the peak strength decreases from 15.82 to 15.68 MPa When flaw inclination angle increases from 45° to 60°, the peak strength increases from 15.68 to 22.54 MPa, and the amplitude of increase is larger (43.8%). Compared with the case of no confining pressure, the peak strength increases by 42.5%. When the lateral stress is 1 MPa, the flaw inclination angle increases from 30° to 45°, and the peak strength increases from 16.96 to 20.7 MPa.When the flaw inclination angle increases from 45°to 60°, the peak strength increases from 20.7 to 23.76 MPa, the amplitude of increase is 14.8%, and the relative confining pressure is 0, when the amplitude of increase is 22.1%. when the flaw inclination angle increases from 45° to 60°, the peak intensity increases from 20.7 to 23.76 MPa. The peak strength increases by 40.1%, it can be seen that under the same lateral stress, the peak strength rock-like materials is increases with the increase of flaw inclination angle, and with the increase of lateral stress under the same flaw inclination angle, the peak strength of specimen has also an increasing trend. This conclusion is consistent with the findings reported of this literature.

## Influencing factors of crack propagation

### Influence of flaw inclination angle on crack propagation

As shown in the Table 5, when α = 30°, the inner tip of the prefabricated flaw begin arises micro-crack, and along with the generation of the tensioned airfoil crack, the upper crack and the lower crack in the tip of the crack extend downward and upward, respectively. The outer tip cracks of the upper and lower cracks extend to the upper boundary and the lower boundary of the specimen respectively. With the continuous increasing of axial stress, shear cracks begin to appear near the inner tip of the upper fracture, and begin to expand along the irregular shape. At the same time, the lower cracks was caused by minor dislocation. There are coplanar shear cracks on the surface of the lower fissures which were coplanar with the lower fissures. The fracture of the rock bridge between cracks was completed at the form of an approximate straight line (Y-1 type penetration), which intensifies the failure of the specimen when the crack at the outer tip extends to the edge of the specimens, and the instability of the specimened was caused by the extension of the crack at the outside tip to the boundary of the specimen.

**Table 5:** Influence of crack inclination on crack propagation (Y: Wing crack; J: Shear crack).

Lateral stress	0.5 MPa	0.5 MPa	0.5 MPa
β	60°	60°	60°
α	30°	45°	60°
Specimens			
Crack propagation			

When α = 45°, the crack propagation process was different from that of the α = 30° specimen, where the two cracks at the tip of the two fractures do not overlap at the rock bridge, but with the inner tip of the other crack. Two airfoil cracks were completed in the rock bridge region in a "fish-hole" shape (Y-2). Finally, when two airfoil cracks at the outer tip of the crack extend to the boundary of the specimens, the specimens was unstable and destroyed.

When α = 60°, the fracture propagation process was similar to that at α = 30°(Y-1 type transfixion).

Combined with the above analysis, when the lateral stress and the rock bridge inclination angle were constant, with the increase of the prefabricated flaw inclination angle, the crack propagation law of the specimen is as follows: there are four cracks initiation and propagation near the tip of the prefabricated flaw, the rock bridge was broken by one cracks, and the rock bridge was broken by two cracks, one of which is dominated by one cracks. The failure modes of the specimens were mainly airfoil tensile failure and fracture. The angle of inclination has no effect on the failure mode of the specimen, and the other results were similar to that of the lateral pressure of 0.5 Mpa, β = 60°.

### Influence of Rock Bridge ligament angle on crack propagation

As shown in the Table 6. When β = 0°, the initial crack begins to occur in the form of tension at the inner tip and the outer tip of the two precast cracks, in which the crack near the outer tip of the upper crack extends to the upper boundary of the specimen. The inner tip extends towards the lower boundary of the specimen. With the increasing of axial stress, shear failure begins to occur in the rock bridge between the two prefabricated flaw, resulting in an irregular shear fracture. The striations are intersected between the rock bridges and accompanied by shedding of fragments. At the same time oblique shear cracks appear near the outer tip of the lower cracks and propagate to the left boundary of the specimens. A coplanar shear crack (mode J) is formed between the rock bridge and the prefabricated double flaw, and the shear crack appears later. Finally, when the wing crack extends to the boundary of the specimens, the specimens loses stability.

**Table 6:** Influence of Rock Bridge inclination on crack propagation (Y: Wing crack; J: Shear crack).

Lateral stress	1.0 MPa	1.0 MPa	1.0 MPa	1.0 MPa
α	30°	30°	30°	30°
β	0°	30°	60°	90°
Specimens				
Crack propagation				

When β = 30°, the failure process of the specimen was similar to that of the rock bridge inclination angle was 0°, and the rock bridge was connected by a coplanar shear crack (J mode) coplanar with the prefabricated double flaw.

When β = 60°, the tensile failure occurs near the outer tip of the upper prefabricated flaw at the beginning of the loading process and the airfoil crack propagates along the curve to the upper boundary of the specimen. At the same time, an airfoil crack propagates to the lower boundary of the specimen near the outer tip of the prefabricated flaw, and the inner tip also produces an inner crack propagating along the inner tip of the prefabricated flaw. With the increasing of axial stress, shear failure begins to occur near the inner tip of the upper prefabricated flaw and a shear cracks was produced. The tangent crack propagates downward and overlaps the whole rock bridge with the airfoil crack produced by the tip of the lower prefabricated flaw in the rock bridge. At this time, the rock bridge is penetrated by mixed crack of airfoil shear (JY-1 mode). Because the shear crack lags slightly behind the airfoil crack, the lap position is closer to the inner tip of the upper precast prefabricated flaw. Finally, the fissures near the outer tip extend to the boundary of the specimen, and the specimen was lose stable.

When β = 90°, the upper prefabricated flaw and the lower prefabricated flaw appear tensile failure near the outer tip of the prefabricated flaw respectively, and propagate to the upper and lower boundary of the specimen respectively. At the same time, an airfoil crack propagates downwards near the inner tip of the upper prefabricated flaw, and a wing crack propagates upward near the inner tip of the lower prefabricated flaw. With the increasing of axial stress, a transverse shear crack is produced near the outer tip of the lower prefabricated flaw, which extends to the left edge of the specimen along an irregular direction (the shape was roughly concave). The prefabricated flaw inner tip cracks do not overlap within the rock bridge, but stagger each other until intersecting with the precast cracks, the rock bridges are perforated in the range of rock bridges. In this case, the rock bridges were intersected with a biplane cracks (Y-3 type), and the biplane cracks and the two cracks were closed to form an approximate quadrilateral shape. Finally, when the fender crack extends to the boundary of the specimens, the specimens was lose stable.

Combined with the above analysis, when the prefabricated flaw inclination angle and lateral pressure were constant, with the increasing of rock bridge inclination angle, the crack propagation law of the specimen was as follows: shear crack through failure mode and shear airfoil tensile crack mixed through failure mode and airfoil tensile crack through failure mode. The number of cracks through is gradually changed from single crack to double crack, and the results are similar to that of lateral stress of 1.0 Mpa, α = 30°.

### Influence of lateral stress crack propagation

As shown in the Table 7, when the lateral stress was 0 MPa, the loading mode of the specimens was uniaxial compression, and the airfoil cracks occur in the form of tensile failure in the vicinity of the inner and outer tip of the two prefabricated flaw. With the increasing of axial stress, the airfoil crack continues to propagate and pass through the rock bridge, and then the wing crack near the outer tip extends to the upper and lower boundaries of the specimen respectively, and the specimen was unstable and destroyed. The failure crack of the specimen was mainly airfoil crack, the shear crack was slightly developed or undeveloped, and the failure mode was tension-type failure. When the lateral pressure was 0.5 Mpa, it was similar to the lateral pressure of 0 Mpa. The failure crack was mainly airfoil crack. Although the shear crack has developed gradually, the diffusion was not obvious. Shear failure occurs only in individual position, and the whole failure mode of the specimen was tensile failure.

**Table 7:** The effects of lateral pressure on crack propagation (Y: Wing crack; J: Shear crack).

α	30°	30°	30°
β	60°	60°	60°
Lateral stress	0 MPa	0.5 MPa	1.0 MPa
Specimens			
Crack propagation			

When the lateral stress was 1.0 Mpa, the airfoil crack appears in the form of tensile failure near the inner tip of the lower prefabricated flaw and the outer tip of the upper prefabricated flaw, and shear failure occurs near the inner tip of the upper fracture with the increasing of axial stress. The diagonal shear crack along the direction of the rock bridge and the coplanar shear crack coplanar with the upper prefabricated flaw extend to the left boundary of the specimen. Finally, the airfoil crack near the outside tip of the specimen extends to the boundary of the specimen, and the specimen is unstable and destroyed. Shear failure occurred in some parts of the specimen, and the shear crack had developed and expanded in a large area, but tensile failure still dominated, and the whole fracture of the specimen was broken. The failure way was tensile shear failure.

Combined with the above analysis, when the prefabricated flaw inclination angle and the rock bridge inclination angle were constant, with the increasing of lateral loading stress, the extension crack of airfoil decreasing, but the shear failure crack increasing obviously. The failure law of the specimen was as follows: airfoil-tensile crack failure and airfoil-shear mode crack failure, and the results were similar to those of side α = 30°, β = 60°.

### Dynamic evolution process of rock crack based on acoustic emission plane positioning

As shown in the Table 8, the dynamic evolution progress of different rock bridge inclination angle for 45° flaw inclination angle at different loading stages. The acoustic emission plane localization results of fracture rupture at β = 0°, 30°, 60°, 90° were similar roughly, α = 45°, β = 0° was select as an examples to analyze the dynamic evolution of rock cracks based on the acoustic emission plane localization.

**Table 8:**
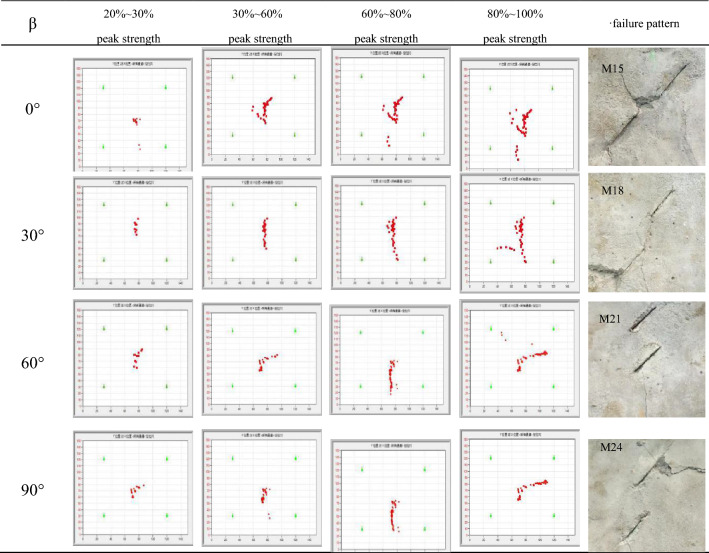
α = 45° crack process of acoustic emission plane localization results.

When the initial loading was 20–30% of the peak stress of the specimen (as shown in Table 9a), the acoustic emission positioning events inside the specimen were small because the internal structure did not form large damage, and the acoustic emission events were mainly caused by the original defects and micro-crack compression of the specimens. When the loading stress reaches 30–60% of the peak stress of the test specimens (as shown in Table 9b), the number of AE location events began to increasing, and mainly distributed in near the center of the image, This is because the internal structure of the rock has begun to produce damage and destroy, and the damage location is mainly concentrated on the center of the specimens. Therefore, it can be inferred that the axial stress is mainly borne by the rock bridge ligament angle between the two prefabricated flaws at this time.

**Table 9:**
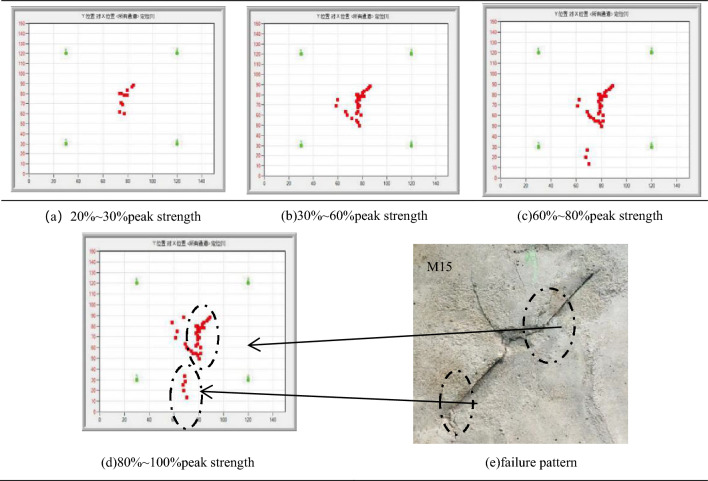
α = 45°, β = 0° Crack process of acoustic emission test Plane localization result.

As the axial stress increasing further, when the loading stress reaches 60–80% of the peak stress of the specimens (as shown in Table 9c), the internal acoustic emission positioning event of the specimens count began to spread transverse or vertically, the test specimens enters the plastic deformation stage, the specimen surface began to produce the macroscopic crack and transverse or longitudinal expansion, the macroscopic crack expansion position and morphology roughly match the image located by the acoustic emission event point. Finally, when the axial stress approaches and reaches the peak stress (as shown in Table 9d), the count of the acoustic emission events does not have a obvious increasing, the internal structure of the specimens has been damaged, the cracks were completed, and the instability was occurs.

Through the technology of acoustic emission plane positioning, the dynamic evolution of the crack during the specimen loading, combined with the dynamic image captured by the digital camera during the process of loading. with the increasing of axial stress, the stress is mainly concentrated in the central area of the rock bridge between the two precast cracks, explaining the rock bridge part of the first cracks and through the phenomenon. When the rock bridge was connected, the crack was immediately extended to the edge of the specimens, losing its bearing capacity. Since the AE event can monitor the dynamic process of specimen rupture in the real time, the expansion trend of crack instability can be predicted.

## Conclusion

1. The crack propagation modes of rock materials with two flaws under the biaxial compression are as follows: wing crack through mode (Y mode), shear crack through mode (J mode), mixed crack through mode (airfoil shear JY mode), longitudinal extension of crack and transverse shear splitting. When the lateral stress is the same as the flaw inclination angle, the specimen of fractured rock goes through four stages, the stage of prefabricated crack closure, the stage of elastic deformation, the stage of crack generation and propagation (or plastic strengthening), and the stage of residual bearing.
2. The peak strength of fractured rock materials increases with the increase of the flaw inclination angle under the same lateral stress, and the peak strength of specimen increases with the increase of lateral stress under the same flaw inclination angle.
3. With the increase of the rock bridge inclination angle, the failure law of specimen from the failure of shear crack develops into the mixed failure of shear airfoil tension crack, and finally the failure of airfoil tension crack occurs. With the increase of lateral stress, the failure law of specimen the tensile crack of airfoil develops into shear crack of airfoil. The crack inclination mainly changes the crack propagation, but does not affect its failure mode.
4. The counting number of acoustic emission events at the center of the sample is relative large, indicating that the cracks appear in the part of the rock bridge firstly. The cracks of the rock bridge expanding constantly and connecting as the loading increasing. The changes of acoustic emission event counts is consistent with the macroscopic damage forms obtained from the experiments.

## Data Availability

Some or all data, models, or codes that support the findings of this study are available from the corresponding author (zhaoyanlinhnust123@163.com) upon reasonable request.
